# Bogong Moths Are Well Camouflaged by Effectively Decolourized Wing Scales

**DOI:** 10.3389/fphys.2020.00095

**Published:** 2020-02-11

**Authors:** Doekele G. Stavenga, Jesse R. A. Wallace, Eric J. Warrant

**Affiliations:** ^1^Surfaces and Thin Films, Zernike Institute for Advanced Materials, University of Groningen, Groningen, Netherlands; ^2^Research School of Biology, Australian National University, Canberra, ACT, Australia; ^3^Lund Vision Group, Department of Biology, Lund University, Lund, Sweden

**Keywords:** *Agrotis infusa*, coloration, wing patterning, melanin, reflectance spectra, scale anatomy

## Abstract

Moth wings are densely covered by wing scales that are assumed to specifically function to camouflage nocturnally active species during day time. Generally, moth wing scales are built according to the basic lepidopteran Bauplan, where the upper lamina consists of an array of parallel ridges and the lower lamina is a thin plane. The lower lamina hence acts as a thin film reflector having distinct reflectance spectra that can make the owner colorful and thus conspicuous for predators. Most moth species therefore load the scales’ upper lamina with variable amounts of melanin so that dull, brownish color patterns result. We investigated whether scale pigmentation in this manner indeed provides moths with camouflage by comparing the reflectance spectra of the wings and scales of the Australian Bogong moth (*Agrotis infusa*) with those of objects in their natural environment. The similarity of the spectra underscores the effective camouflaging strategies of this moth species.

## Introduction

Bogong moths (*Agrotis infusa*) are night-flying moths, well-known for their highly directional, biannual long-distance seasonal migrations to and from the Australian Alps (Common, 1954; Warrant et al., 2016), a feat which requires the Earth’s magnetic field and visual landmarks as navigational cues (Dreyer et al., 2018). The adults aestivate over the summer in cool alpine caves in the mountains of southeast Australia, including the Brindabella Ranges of the Australian Capital Territory, the Snowy Mountains of New South Wales and the Bogong High Plains in Victoria. Following this period of aestivation, the same individuals that arrived in the mountains months earlier leave the caves and return to their breeding grounds (a journey of up to 1000 km) to mate, lay their eggs and die. The Bogong moth is culturally important for the Aboriginal people, who used the moths as a significant source of protein and fat during their annual summer gatherings in alpine areas (Flood, 1980, 1996).

The moths’ name, bogong, is derived from the Aboriginal word bugung, meaning “brown moth” (from the extinct Dhudhuroa language of northeast Victoria), which aptly describes their coloration. The moth’s brown colors presumably serve background matching (Stevens and Merilaita, 2009), i.e., to make the moths inconspicuous to other predators during the day, both during their migration across the plains and following arrival in the mountains (when they can be preyed upon by the Mountain pygmy possum (Gibson et al., 2018) and various species of birds, among other predators). The classical example of camouflage is the peppered moth (*Biston betularia*), which developed a darker wing scale color as trees became covered in industrial soot and via the forces of natural selection induced by predatory birds (Majerus et al., 2000; Cook et al., 2012). Light-colored moths, resting in the daytime on dark-brown trees, are easily recognizable prey, so that dark-brown colored moths have a higher chance of survival.

The color of lepidopteran insects (moths and butterflies) is determined by the lattice of scales that cover the wings like shingles on a roof (Nijhout, 1991; Kinoshita, 2008). The chitinous wing scales are organized into two laminae, i.e., a thin, more or less flat lower lamina, and a highly structured upper lamina which consists of rows of parallel ridges with inter-linking cross-ribs that leave more or less open windows. The upper lamina is connected to the lower lamina by trabeculae, pillar-like elements serving as mechanical struts and spacers (Ghiradella, 1991, 1998, 2010).

Commonly, the color of the individual scales has a pigmentary (or chemical) origin. If the scale contains a considerable amount of pigment, incident light reflected and scattered by the scale structures is then selectively filtered, dependent on the pigment’s absorption spectrum, so that a distinct pigmentary color remains. Various pigment classes are expressed in lepidopteran wing scales. Pterins are the pigments generally encountered in pierid butterflies (Descimon, 1975; Wijnen et al., 2007), kynurenine and its derivatives, the ommochromes, are generally identified in nymphalids (Nijhout, 1997; Wilts et al., 2017), and the papilionids are colored by papiliochrome pigments (Umebachi, 1985; Nijhout, 2010; Nishikawa et al., 2013; Wilts et al., 2014). Some papilionids, as well as the Geometrinae and other moths, use blue-green bile pigments, neopterobilins (Choussy et al., 1973; Barbier, 1986). Within the Geometrinae a novel green pigment type, geoverdin, has also been reported (Cook et al., 1994). But undoubtedly the commonest pigment found in the Lepidoptera, and certainly in moths, is melanin, a broadband-absorbing pigment responsible for most brown and black colors.

A wing scale’s color can also have a structural (or physical) basis. Regularly arranged, nanosized scale structures cause wavelength-dependent light interferences, as for instance is the case in intense blue *Morpho* butterflies, where the building blocks of the scale ridges, the lamellae, form a stack that functions as an optical multilayer (Vukusic and Sambles, 2003; Giraldo et al., 2016). The lower lamina can also create a structural color, because its thickness is of the order of 100–200 nm. It hence acts as an optical thin film, causing a distinct reflectance in a restricted wavelength range, critically depending on the precise thickness of the thin film and angle of illumination or view. The lower lamina can thus determine the scale color in the specific case that the scale is unpigmented (Stavenga et al., 2014). The highly convoluted upper lamina commonly acts as a broad-band light diffuser that spreads light in a wide spatial angle, resulting in less angle dependence of the iridescent colors (e.g., Stavenga et al., 2014).

The anatomical organization and pigmentation of the scales and wing colors resulting from the lattice of scales have been investigated in only a few moth species, and mostly in those featuring conspicuous colors, as in the extremely colorful day-flying swallowtail moths *Urania fulgens* and *Urania leilus* (Onslow, 1923; Süffert, 1924; Lippert and Gentil, 1959). A particularly exuberantly colored species is the sunset moth *Chrysiridia ripheus* (Uraniinae), which applies advanced multilayer optics in sometimes strongly curved scales, resulting in distinct color mixing and polarization effects (Yoshioka and Kinoshita, 2007; Yoshioka et al., 2008). The scales of the basal moth *Micropterix aureatella*, which like the diurnally active uraniines is a day-flying moth, have a fused upper and lower lamina. The thickness of the resulting single thin film varies slightly, thus causing metallic gold, bronze and purple colors (Kilchoer et al., 2018). The similarly day-active palm borer moth *Paysandisia archon* has brownish forewings with prominently orange colored hindwings due to a cover of ommochrome-pigmented scales (Stavenga et al., 2018). The nocturnal moth *Eudocima materna* (Noctuidae) has similarly ommochrome-pigmented hindwings with sparkling effects caused by patches with mirror scales (which have a thin film reflector in their lower lamina; Kelley et al., 2019). However, moths are generally dull colored, due to high concentrations of melanin in their scales, presumably for suppressing possible structural coloration. Here we focus on the Bogong moth, to investigate how dull-brown moths organize their appearance by variously expressing melanin in their wing scales.

## Materials and Methods

### Specimens

Bogong moths used in this study were bred in Lund from adults captured in Australia. We furthermore collected pieces of granite from a Bogong moth aestivation cave (South Rams Head, Kosciuszko National Park, elevation 1860 m), as well as a variety of bark samples taken from: Coastal tea-tree (*Leptospermum laevigatum*), River bottlebrush (*Callistemon sieberi*), Crimson bottlebrush (*Callistemon citrinus*), River oak (*Casuarina cunninghamiana*), Brittle gum (*Eucalyptus mannifera*), Argyle apple (*Eucalyptus cinerea*), Silver-leaved ironbark (*Eucalyptus melanophloia*), Mountain gum (*Eucalyptus cypellocarpa*), Scribbly gum (*Eucalyptus rossii*), Snow gum (*Eucalyptus pauciflora*), Weeping snow gum (*Eucalyptus lacrimans*), Narrow-leaved Sally (*Eucalyptus moorei*), Parramatta red gum (*Eucalyptus parramettensis*), and *Eucalyptus* sp. aff. *notabilus*. These tree species were chosen because they are likely to be encountered along the migratory route as well as upon arrival in sub-alpine areas where moths feed for some weeks before ascending to their aestivation caves at higher elevation (Supplementary Figure S1 and Table 1). We have not given attention to sexual differences, as there is no marked sexual dimorphism. We have to note that the moths do come in many shades of brown; particularly males can (although not always) be much lighter in color than females.

**TABLE 1:** The distributions of Australian trees species used for bark spectra measurements (Supplementary Figure S1) and their likelihood of being encountered by migrating Bogong moths.

Species	Likely resting spot?	Notes^1^
*Eucalyptus* sp. aff. *notabilus*	No	Distribution is predominantly east of the Great Dividing Range
*Leptospermum laevigatum*	No	Distribution confined to coastal habitats. Bogong moths may be found here, but it is not where most are coming from
*Eucalyptus moorei*	No	Distribution is predominantly east of the Great Dividing Range
*Eucalyptus parramattensis*	No	Relatively localized around the Sydney region
*Eucalyptus mannifera*	**Yes**	Widespread and abundant in south-eastern NSW
*Callistemon sieberi*	**Yes**	Widespread and abundant in eastern NSW
*Callistemon citrinus*	No	Distribution mostly east of Great Dividing Range
*Casuarina cunninghamiana*	**Yes**	Widespread and abundant across eastern NSW and QLD
*Eucalyptus cinerea*	**Yes**	Locally abundant just north of the NSW Snowy Mountains; also occurs in VIC
*Eucalyptus melanophloia*	No	Distributed across northern NSW as well as QLD
*Eucalyptus cypellocarpa*	No	Occurs in wet forest in sheltered valleys, eastern slopes of the Great Dividing Range
*Eucalyptus rossii*	**Yes**	Widespread and abundant in eastern NSW, although these would not provide adequate hiding places for Bogong moths due to their light color; another moth, *Ogmograptis scribula*, in contrast, highly favors them
*Eucalyptus pauciflora*	**Yes**	Widespread and dominant across eastern NSW, and throughout VIC and TAS (occurs in alpine areas); Bogong moths have been observed feeding on them
*Eucalyptus lacrimans*	**Yes**	Subalpine species, restricted to the Adaminaby region of the NSW Snowy Mountains

*^1^ NSW, New South Wales; VIC, Victoria; QLD, Queensland; TAS, Tasmania.*

### Scanning Electron Microscopy

Wing pieces and isolated scales were prepared for scanning electron microscopy by sputter-coating with gold (Cressington 108 auto, 45 s, 20 mA). The preparations were viewed using a scanning electron microscope (SEM; Hitachi SU3500) at 5 kV.

### Spectrophotometry

Reflectance spectra of different areas of the Bogong moth wings, as well as of the granite and bark samples, were measured with a bifurcated reflection probe (Avantes FCR 7-UV-200), using an AvaSpec 2048-CCD detector array spectrometer (Avantes, Apeldoorn, Netherlands). The light source was a deuterium-halogen lamp (AvaLight-D(H)-S), and the reference was a white diffuse reflectance tile (Avantes WS-2). Reflectance spectra of isolated scales, attached to a glass micropipette using Bison glass kit (hardening under UV light), were measured with a microspectrophotometer (MSP). The MSP was a Leitz Ortholux microscope (Leitz, Wetzlar, Germany) with an Olympus 20× objective, NA 0.46 (Olympus, Tokyo, Japan). A xenon arc lamp was used as a light source. The area measured with the MSP was a square with edge length 5–10 μm, determined by a square diaphragm in the microscope’s image plane, which was in turn imaged at the entrance of an optical fiber connected to the detector array spectrometer; the white diffuse reflectance tile was also here used as a reference. Due to the glass optics in the microscope, the MSP spectra were limited to wavelengths >350 nm. Absorbance spectra of isolated scales were also measured with the MSP, while the scales were immersed in immersion oil (*n* = 1.515) in order to reduce scattering.

### Imaging Scatterometry of Single Wing Scales

For investigating the spatial reflection characteristics of the scales, we performed imaging scatterometry (Stavenga et al., 2009; Wilts et al., 2009). A scale attached to a glass micropipette was positioned at the first focal point of the ellipsoidal mirror of the imaging scatterometer. The scatterograms were obtained by focusing a white light beam with a narrow aperture (<5°) at a small circular area (diameter 13 μm), and the spatial distribution of the far-field scattered light was then monitored. The exposure times of the scatterograms were appropriately adjusted so as to obtain an image of maximal contrast.

### Modeling the Reflectance Spectra of Thin Films

The reflectance spectra of chitinous optical thin films in air were calculated for normally incident light using an expression derived from the classical Airy formula (Stavenga, 2014).

## Results

### Wing Colors and Reflectance Spectra

**FIGURE 1:**
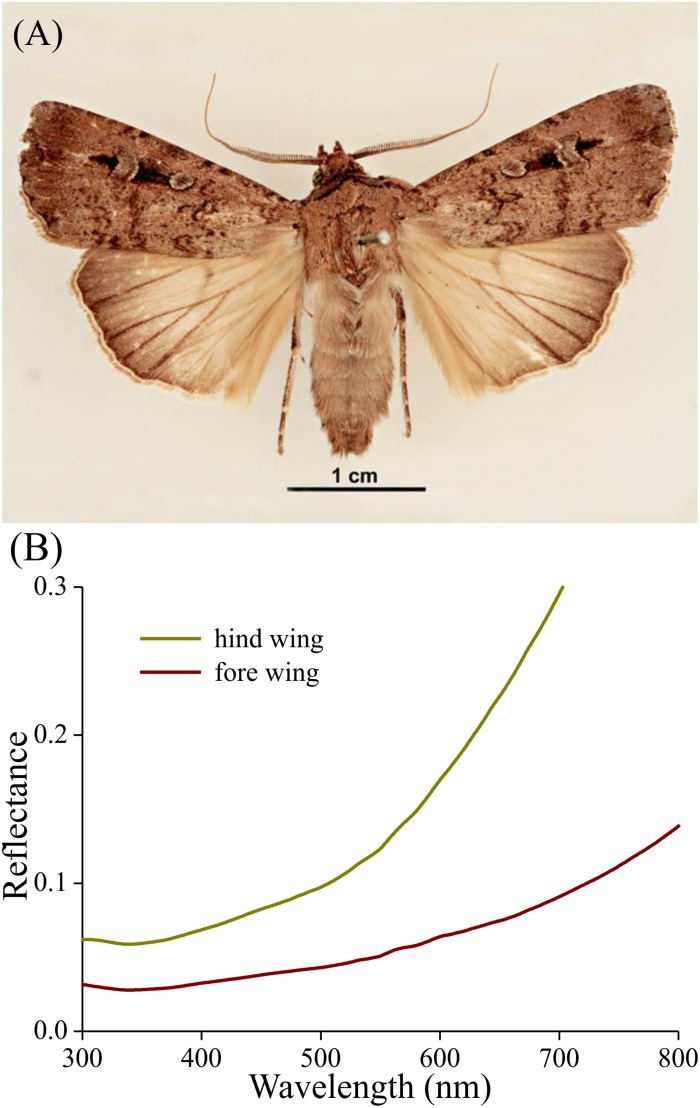
Bogong moth coloration. **(A)** A pinned specimen with exposed fore and hind wings (from http://www.padil.gov.au/pests-and-diseases/pest/main/136308/5837#; source Lucinda Gibson and Ken Walker, Museum Victoria, Australia). Image available for free use under a Creative Commons Attribution 3.0 Australia License, CC BY 3.0 AU. **(B)** Reflectance spectra of the upper sides of the hind and fore wing measured with a bifurcated reflection probe.

Bogong moths have brown bodies and wings. The dorsal forewings are dark-brown with black patches, which cover (when resting) the more or less uniformly light brown dorsal hindwings and the intermediate brownish thorax and abdomen (Figure 1A). The ventral wing edges are light brown. As an example of the differences in saturation, Figure 1B shows reflectance spectra of local areas of the dorsal fore- and hindwing. In both cases, the reflectance increases monotonically with increasing wavelength, which is characteristic of a melanin-pigmented tissue (Figure 1B).

### Scale Structures

**FIGURE 2:**
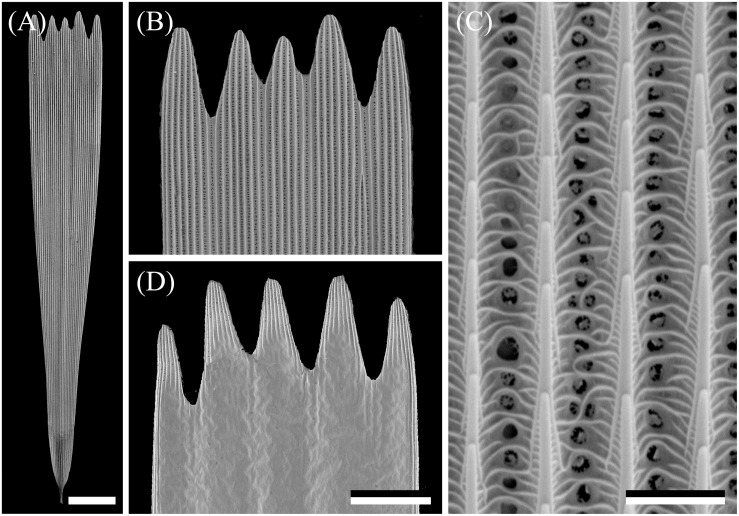
Scanning electron micrographs of a forewing scale. **(A)** An isolated scale with dented tip area and on the other end the root. **(B)** Magnified view of the abwing (upper) side of the tip area of **(A)**. **(C)** The scale ridges, consisting of slightly overlapping lamellae and connected by cross-ribs, which leave minor open windows. **(D)** The adwing (under) side of a tip area. Scale bars: **(A)** 50 μm, **(B,D)** 25 μm, **(C)** 2.5 μm.

The wings have a cover of numerous scales, and thus the wing coloration will be determined by how the scales reflect incident light. The primary determinant of the scale reflectance is melanin pigment, as suggested by Figure 1B. Yet, a crucial contributing factor can be the scales’ fine-structure, which was investigated by performing scanning electron microscopy. The size of the wing scales appears to be rather variable, but the structure adheres to the classical format. The upper side is an array of ridges that are connected by cross-ribs, which leave minor open windows (Figures 2A–C), and the underside features a more or less flat, slightly wrinkled plane (Figure 2D).

The ridges of lepidopteran wing scales consist of overlapping lamellae, but the overlap of the ridge lamellae in Bogong moth scales is very minor, so that a possible structural coloration of the scales’ upper lamina can be neglected. As is universally the case in lepidopteran scales, the lower lamina will act as a thin film reflector, and thus some structural coloration contributions can be expected.

### Scale Pigmentation, Reflection, and Scattering

**FIGURE 3:**
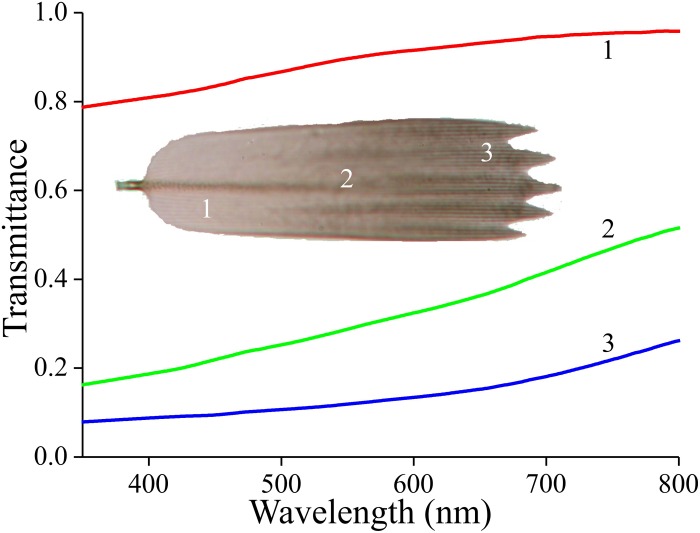
Transmittance spectra of an isolated forewing scale immersed in immersion oil, in the root (1), middle (2), and tip (3) area, measured with a microspectrophotometer.

To first check whether the scales contain a melanin-like pigment, we performed transmission-microspectrophotometry on single scales immersed in oil (Figure 3). At all scale locations, the transmittance increased monotonically with increasing wavelength, which is indeed the hallmark of melanin. The transmittance strongly varied along the length of the scale, however. Near the root, the transmittance was high, i.e., at this location the scale is almost transparent (Figure 3, #1), but near the tip the transmittance was hardly more than ∼10% across the whole visible wavelength range (Figure 3, #3).

**FIGURE 4:**
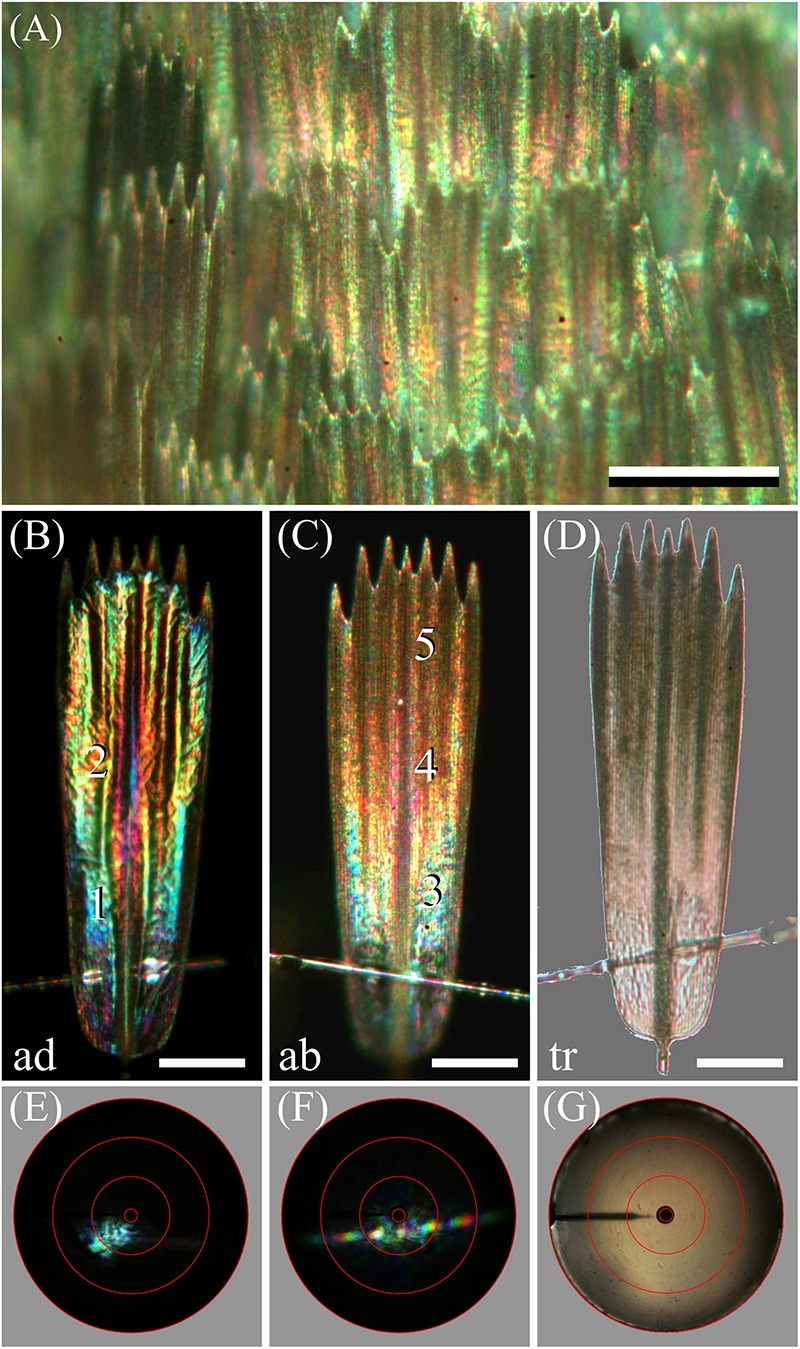
Spectral and spatial characteristics of Bogong moth scales. **(A)** Epi-illumination micrograph of the scale pattern, locally in the forewing. **(B)** Epi-illumination of the adwing side of an isolated forewing scale glued to a glass micropipette. **(C)** Same as **(B)** except for the abwing side. **(D)** Same as **(B)** but with transmitted light. **(E)** Scatterogram of area 1 of **(B)** with narrow-aperture illumination. **(F)** Scatterogram of area 4 of **(C)** with narrow-aperture illumination. **(G)** Scatterogram of area 4 of **(C)** with wide-aperture illumination. Scale bars: **(A)** 100 μm, **(B–D)** 50 μm. The red circles in **(E–G)** indicate reflection angles of 5°, 30°, 60°, and 90°. For reflectance spectra measured at locations numbered 1–5, see Figure 5.

How this steep gradient in melanin pigmentation might affect scale coloration was then investigated by epi-illumination microscopy. This revealed that the coloration of the lattice of scales on the forewing is far from uniform (Figure 4A). When observing isolated, single scales, the diverse coloration was particularly apparent, as shown in the example of Figures 4B-D. The under (or adwing) side features a diverse color pattern, from blue near the root to purplish in the center and tip area, which indicates that the lower lamina of the scales, acting as a thin film reflector, has a variable thickness (Figure 4B). The upper, abwing side is blue-greenish at the root but reddish-brown at the tip (Figure 4C). In transmitted light, the scale showed a dull, brown color, as expected from the scale’s melanin pigmentation (Figure 4D).

The color differences in the reflection patterns obtained from both scale sides is readily explained from the gradient in pigmentation. The adwing bottom area, near the root (Figure 4B), has a similar blue-green color as the abwing bottom area (Figure 4C), although the color of the latter is slightly more diffuse. The explanation is similar to that of the coloration of a transparent, unpigmented scale (Stavenga et al., 2014). When incident light enters an unpigmented scale from above, from the abwing side, a major fraction of it will pass the upper lamina and reach the lower lamina, where it will be partly reflected, depending on the thin film’s reflectance spectrum. A major fraction of this reflected light will then pass again to the upper lamina, and thus, in the absence of a pigment filter, the color of the reflected light will be equal to the color of the adwing reflected light.

However, the middle and tip areas of the upper lamina do contain a major amount of melanin pigment – these areas will then act as a gradual spectrally long-pass brown filter. Consequently, incident light that enters the pigmented scale areas from the abwing side will suffer from the pigment filter before it reaches the lower lamina. There it is subsequently reflected, and the resulting light flux will have to pass again through the pigment filter on its way back before it leaves the scale at the abwing side. An increasingly brown color thus results, proportionally with the pigment density (Figures 4C,D).

We further investigated the structural coloration by applying imaging scatterometry, using a narrow aperture illumination beam. The scatterogram of a blue area on the adwing side featured a local blue spot, meaning a strongly directional, spatially restricted reflection, confirming the action of a specular thin film reflector (Figure 4E). On the other, abwing side, of the scale, the scatterogram showed a striking diffraction pattern (Figure 4F). This can be immediately understood, because the upper lamina of the scales consists of an array of parallel ridges, which acts as a diffraction grating. We finally applied a wide-angled illumination beam to the abwing scale side (central area #4 of Figure 4C), which yielded the scatterogram of Figure 4G. Clearly, the cumulative diffraction patterns created by the large-aperture illumination then superimpose, resulting in a diffuse scatterogram. The overall brown color demonstrates that the backscattered light is effectively filtered by the considerable amount of melanin pigment.

**FIGURE 5:**
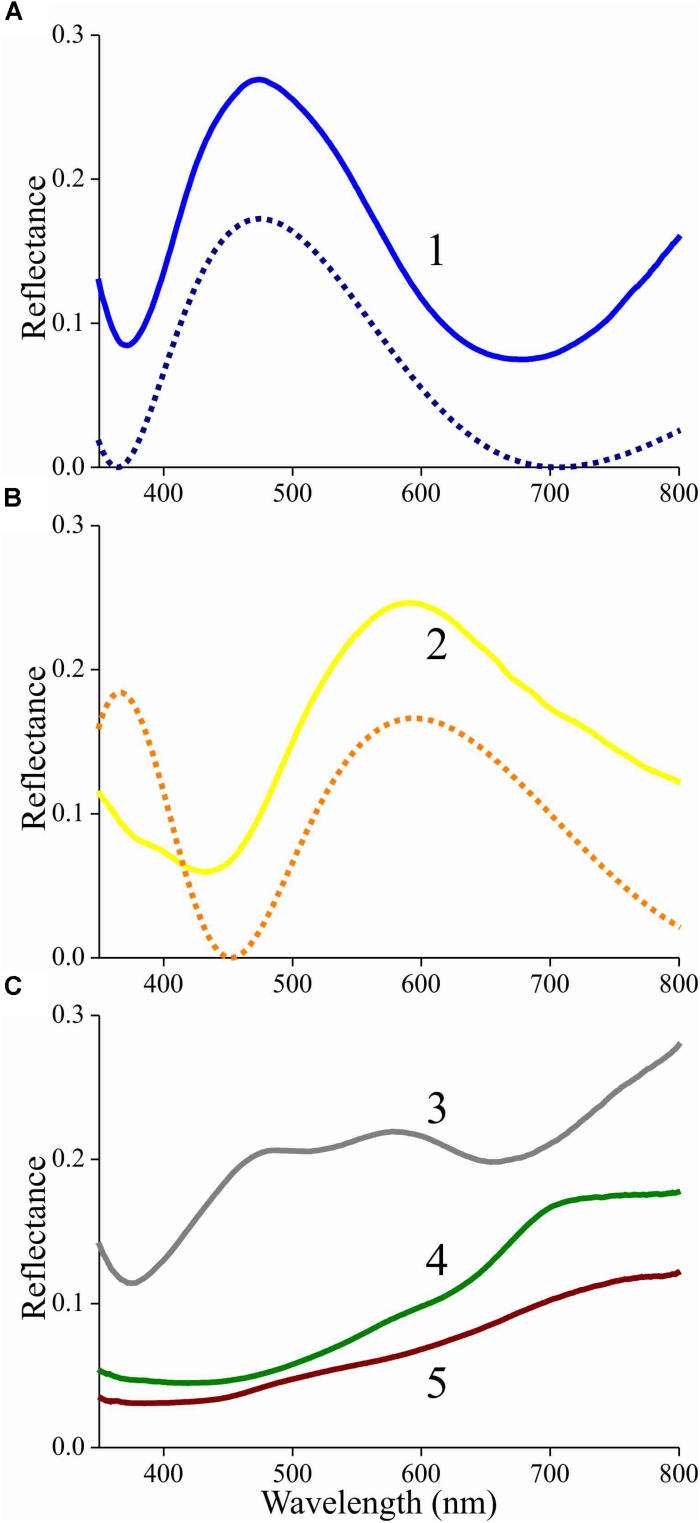
Reflectance spectra of an isolated scale measured with a microspectrophotometer. **(A)** Solid curve: reflectance spectrum of area 1 of Figure 4B (adwing); dashed curve: modeled reflectance spectrum for a chitinous thin film in air with thickness 230 nm. **(B)** Solid curve: reflectance spectrum of area 2 of Figure 4B (adwing); dashed curve: modeled reflectance spectrum for a chitinous thin film in air with thickness 290 nm. **(C)** Reflectance spectra of areas 3–5 of Figure 4C (abwing).

To further ascertain that the lower lamina acts as a thin film, we used a microspectrophotometer to measure reflectance spectra from the lower lamina in locations #1 and 2 of Figure 4B (Figures 5A,B, solid curves). Except for a background offset, the spectra closely resemble the reflectance spectra of thin films with thickness 230 and 290 nm, respectively (Figures 5A,B, dotted curves). The reflectance offset can be easily understood, because a major fraction of the applied light is transmitted by the thin film and subsequently will be partly backscattered by the upper lamina. A large fraction of that light flux will be subsequently transmitted by the lower lamina and thus contribute to the reflected light. Painstaking transmission electron microscopy will be necessary to confirm the derived thicknesses of the scale’s lower lamina.

The reflectance spectra measured from the abwing scale side are rather different (Figure 5C). In the spectrum of the blue-green colored root area (Figure 4C, #3), the contribution of the underlying thin film can be recognized, but the reflectance spectra of the more distal areas (Figure 4C, #4, 5) are dominated by melanin filtering (Figure 5C).

In the scale lattice on the wing, the scales strongly overlap, so that only the most distal part is exposed. Therefore, the reflectance spectra of intact wings (Figure 1B) will have the monotonically rising form characteristic of melanin-pigmented tissue (as seen in Figure 5C, #5). In conclusion, because the tip area is fully exposed in intact moths, the thin film reflections that are intrinsic to the lower lamina are effectively removed in that area by a high concentration of melanin pigment.

### Reflectance Spectra of Granite and Bark

**FIGURE 6:**
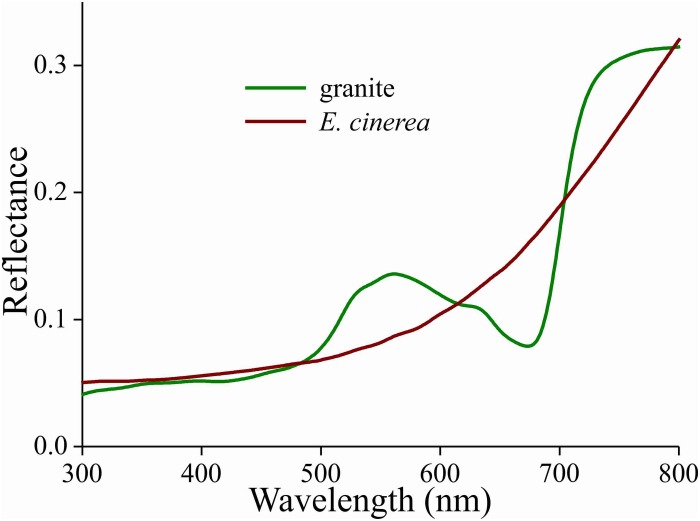
Reflectance spectra of a piece of cave granite and a piece of bark from the Argyle apple tree (*Eucalyptus cinerea*).

**FIGURE 7:**
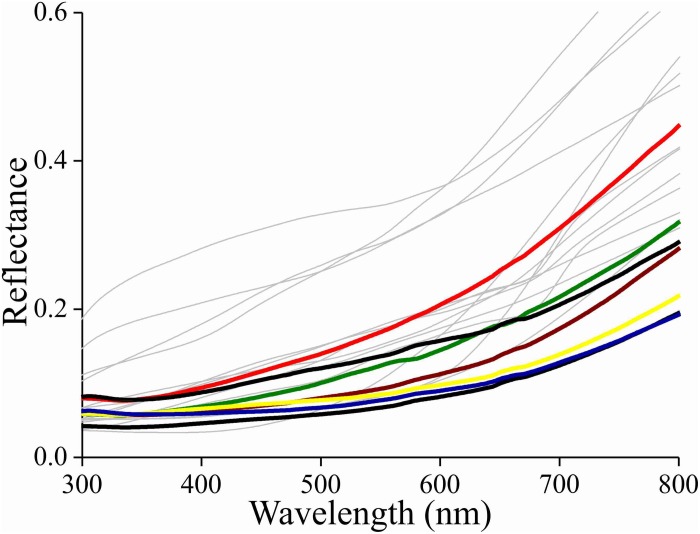
Reflectance spectra of the bark of the trees listed in Table 1 as likely resting spots (thin lines) and reflectance spectra of various places on the forewings of a few Bogong moths (bold lines).

**FIGURE 8:**
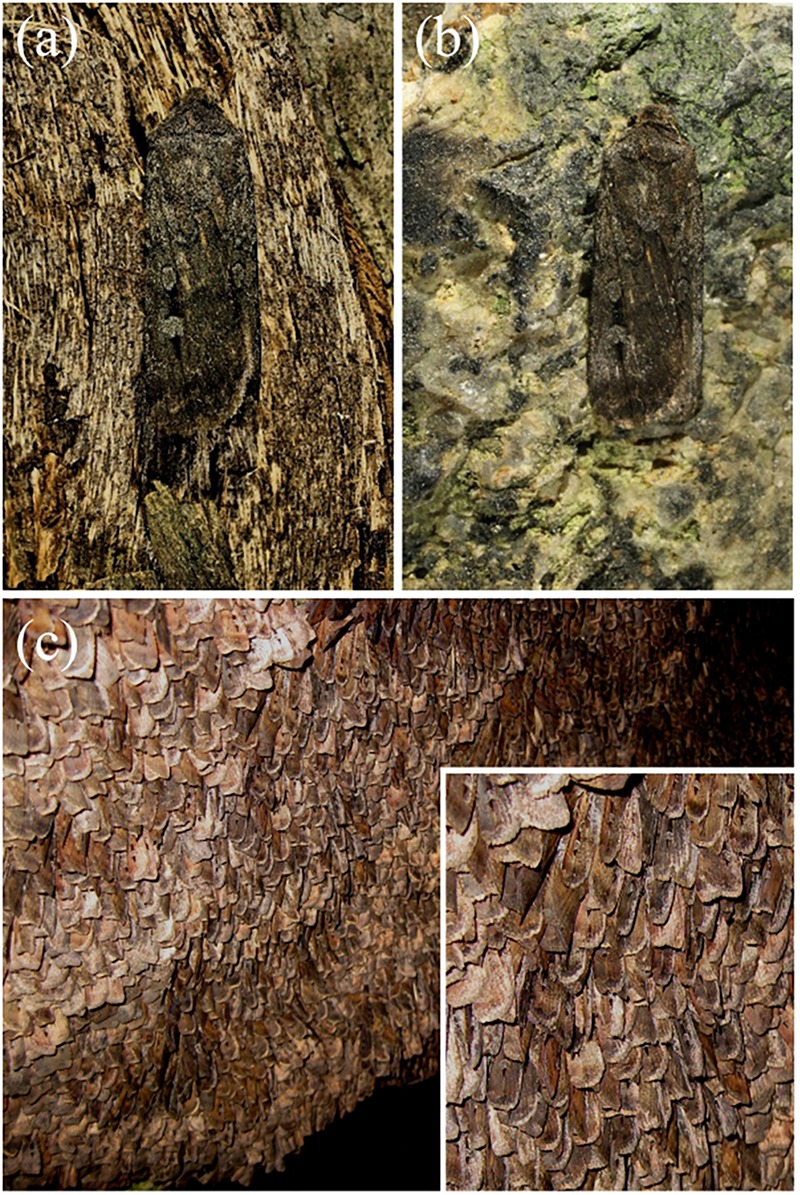
Bogong moth camouflage on bark of the Argyle apple (*Eucalyptus cinerea*) **(a)**, and on a small piece of granite **(b)** taken from the wall of an aestivation cave at South Ramshead in the Kosciuszko National Park, New South Wales (elevation 1860 m). **(c)** Bogong moths aestivating in the same cave from which the granite sample was taken in **(b)**. Inset: close-up. (Photographs taken by EJW on December 23rd 2010).

Due to the melanin pigmentation and its effective blocking of the thin film reflections, the coloration of Bogong moths is not particularly striking, especially on their dorsal forewings. An obvious reason for the dull colors is to achieve suitable background matching when the moth is resting with the forewings covering the body. To underscore this view, we assembled a variety of objects – granite from their aestivation caves and the bark of several trees that are potential resting places for the moths (Table 1) – and measured their reflectance spectra (Figures 6, 7 and Supplementary Figure S1). The reflectance spectrum of the granite has a slight hump in the green part of the spectrum, presumably due to a thin algal coating arising from the moist conditions of the cave, while bark samples from the species of trees tested have a clear brown color. The greenish surface of the cave granite is clearly less well matched to the Bogong moth’s wing color than the trees’ bark, although some degree of camouflage will be nonetheless provided (Figures 6, 8a,b). The reflectance spectra of the bark of trees that are likely resting places vary considerably, but the reflectance spectra of the forewings of the rather dark female Bogong moths we were using vary in the same range (Figure 7). Less darkly-colored specimens will still be able to achieve background matching on trees having higher spectral reflectance. Whether Bogong moths actively select resting places that precisely match the reflectance of the background (as has been noted in other moths, e.g., Sargent, 1966), will be the theme of a future study.

## Discussion

The gradient of melanin expressed in the wing scales of the Bogong moth is very sensible, because the high melanin concentration in the distal, exposed part of the scales filters the reflections of the scales’ lower lamina, so that only the long-wavelength part of the lower lamina’s reflectance spectrum contributes to the total wing reflectance. Several butterfly species do use the lower lamina’s thin film properties to create a distinct scale color, for instance the strikingly iridescent wing scales of the Mother-of-pearl, *Salamis parhassus* (Onslow, 1923) and the pigmentless blue scales of nymphalines (Stavenga et al., 2014). In most butterflies, however, the wing colors are due to pigments, which are expressed in the upper lamina and absorb selectively in a restricted wavelength range, thus resulting in a scale reflectance spectrum in the complementary wavelength range. Remarkably, the reflectance spectra of lower and upper lamina are often tuned, so that their summed reflections create bright scale colors (Stavenga et al., 2015; Wilts et al., 2015). On the other hand, most butterflies also have black scales, frequently marking the wing margin, which is caused by very dense melanin pigmentation that fully suppresses the lamina reflections (e.g., Stavenga et al., 2014).

Butterflies are admired for their striking, colorful appearances, but interestingly, many butterfly species have ventral wings that are darkly colored, for example the nymphaline Peacock butterfly *Aglais io* and the Admiral *Vanessa atalanta* (e.g., Stavenga et al., 2014). When at rest, their wings are closed and then they are camouflaged owing to their inconspicuous ventral coloration. The iconic *Morpho* butterflies have dorsal wings that have a bright-blue structural color, but their ventral wings are only mildly colored by pigmented scales and patterned by modest eyespots. As for *Vanessa*, the ventral wings of *Morpho* will provide camouflage when these butterflies are not on the wing. Other remarkable cases of background matching are offered by some lycaenids: the scales at the ventral wing sides of the Green Hairstreak, *Callophrys rubi*, have highly sophisticated gyroid cuticular structures that create a green reflectance matching that of leaves (Michielsen et al., 2010), and the variable thin films of the ventral scales of the Angled Sunbeam, *Curetis acuta*, act as silvery reflectors that radiate the green colors of the leaves on which the butterflies have landed (Wilts et al., 2013).

In the Bogong moth’s wings, a dense melanin filter blocks the lower lamina reflections and thus prevents the danger of conspicuousness. The correspondence of the moth’s coloration with that of the objects in its habitat underscores the hypothesis that the dull colors of this moth (and others) function for camouflage through background matching (Thayer, 1909; Cott, 1940). The measured reflectance spectra from the bark samples are well matched to the spectra measured from the moth’s wings (Figures 7, 8), thus suggesting that visual camouflage from diurnal predators has been a major selective pressure during the evolution of Bogong moth coloration, and most likely that of many other species of nocturnal moths that need to find concealing shelter during daytime hours (e.g., Thayer, 1909; Cott, 1940; Sargent, 1966). Indeed, many species of moths are even capable of optimizing their orientations and locations on tree bark to maximize this camouflage (Sargent, 1966; Endler, 1984; Kang et al., 2012).

Interestingly, the melanin pigment is deposited preferentially in the tip of the wing scale, suggesting that this is an optimization of production costs. A study on the brown colored-satyrine butterfly *Pararge aegeria* concluded that melanization of the wing scales is indeed costly (Talloen et al., 2004). The wing scales of moths have a high melanin content, the cost of which is necessary to block the thin film reflections of the lower lamina and to obtain a camouflaging brown color. How the pigmentation is locally controlled is an emerging question that is not only intriguing but at the same time complex, since melanin synthesis is co-regulated with scale structure as was revealed in another satyrine, *Bicyclus anynana* (Matsuoka and Monteiro, 2018).

The melanization of the Bogong moths’ wing scales is not uniform in a single scale (Figure 3), but it is also rather variable among the different scales of the wings’ cover (Figure 1A). This serves to give resting moths a mottled appearance, which may add to matching the background of similarly inhomogeneously colored tree bark (Endler, 1984; Kang et al., 2012). Furthermore, the moth’s contours, together with the more or less striped wing patterning, may endow the moths with a potentially disruptive coloration, thus further enforcing camouflage (e.g., Schaefer and Stobbe, 2006; Stevens and Merilaita, 2009). The dark spots on the forewings may act as distractive markings or for surface disruption (Stevens and Merilaita,2009; Cuthill, 2019).

The scales are very densely packed and overlap each other multifold. The dense scale cover may, however, not primarily function for (in)visibility, as it has additional functions. For instance, by providing thermal insulation, the moths can migrate over long distances during cold nights and withstand up to 4 months of dormancy in their high-altitude aestivation caves where nighttime temperatures, even in summer, can fall to several degrees Celsius below zero. The wing scales of moths can also have an acoustical function, by absorbing sound frequencies corresponding to the calls of echo-locating bats, thus reducing echo power (Zeng et al., 2011). Furthermore, scale attachment in sockets on the wings is sufficiently loose that the scales are shed when their owner becomes caught in a spider web, hence allowing the moth to escape (Eisner, 2003).

Whereas the Bogong moths are well matched to a bark background, the color of the granite rock in their caves deviates by not providing as good a match. However, the moths tile tightly (with up to 17,000 individuals per square meter; Common, 1954), thus fully carpeting the cave walls so well and so extensively that the granite cave wall is only seen at the periphery of these moth carpets (Figure 8c). In other words, although the reflectance spectra of granite and moths may somewhat differ, within the interior of such a carpet, the moths are camouflaged against themselves.

## Data Availability Statement

All datasets generated for this study are included in the article/Supplementary Material.

## Author Contributions

DS and EW conceived the project. DS, EW, and JW collected the data. DS wrote the first version of the manuscript. All authors edited the draft until the final version was complete.

## Conflict of Interest

The authors declare that the research was conducted in the absence of any commercial or financial relationships that could be construed as a potential conflict of interest.

## Supplementary Material

FIGURE S1 Reflectance spectra of aestivation cave wall granite and tree bark measured with a bifurcated reflection probe. When the bark color was rather uniform, only one spectrum is shown. Several spectra are included when the surface coloration was varied.
