# Supplementary figures and images for: Bogong Moths Are Well Camouflaged by Effectively Decolourized Wing Scales
Source: Front Physiol. 2020 Feb 11;11:95. doi: 10.3389/fphys.2020.00095 (PMC7026391; doi:10.3389/fphys.2020.00095)

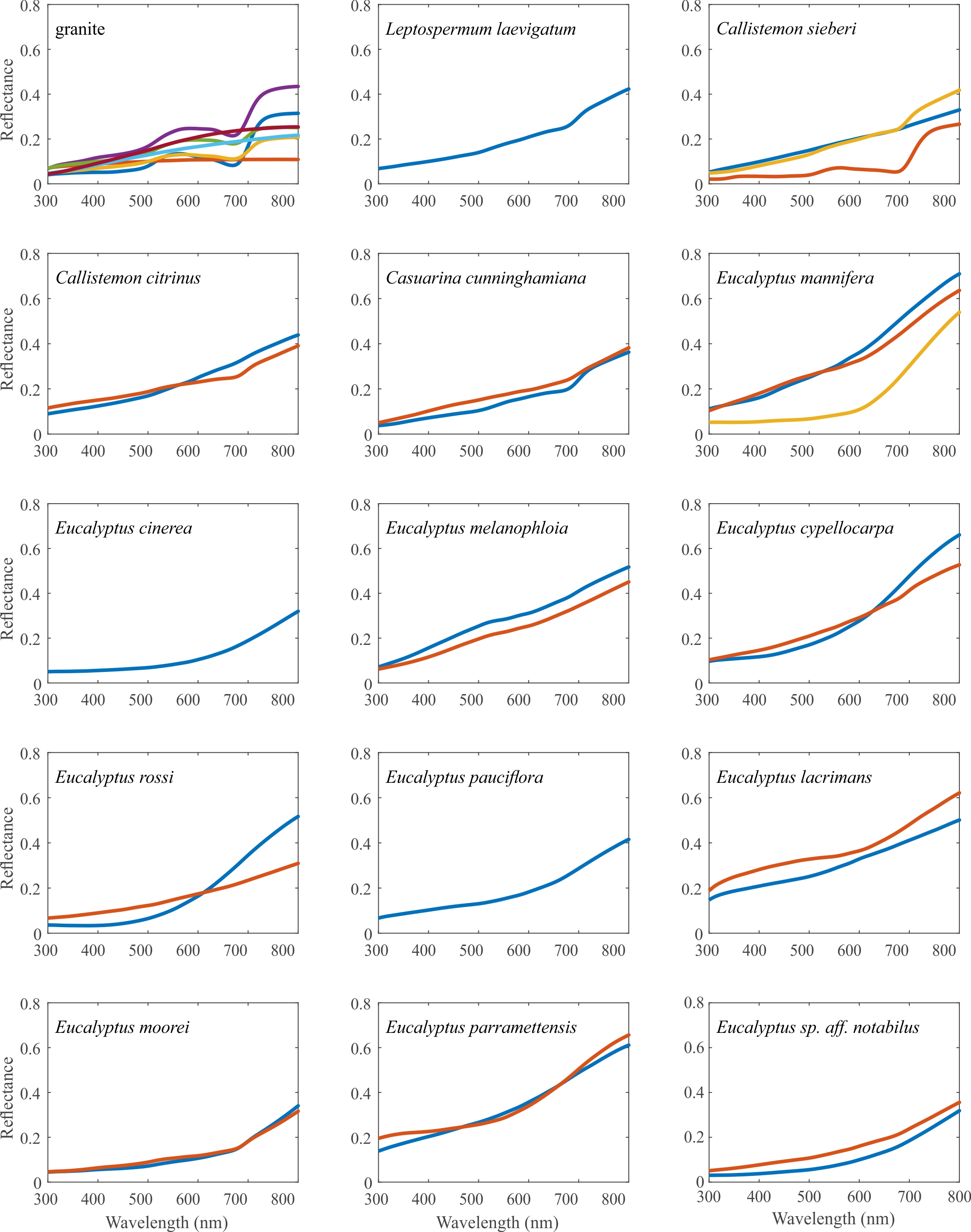

Supplement: FIGURE S1 — Reflectance spectra of aestivation cave wall granite and tree bark measured with a bifurcated reflection probe. When the bark color was rather uniform, only one spectrum is shown. Several spectra are included when the surface coloration was varied. [file Image_1.JPEG]
